# Identifying differentially coexpressed module during HIV disease progression: A multiobjective approach

**DOI:** 10.1038/s41598-017-00090-2

**Published:** 2017-03-07

**Authors:** Sumanta Ray, Ujjwal Maulik

**Affiliations:** 1 Department of Computer Science and Engineering, Aliah University, Kolkata, 700156 India; 2 Department of Computer Science and Engineering, Jadavpur University, Kolkata, 700108 India

## Abstract

Microarray analysis based on gene coexpression is widely used to investigate the coregulation pattern of a group (or cluster) of genes in a specific phenotype condition. Recent approaches go one step beyond and look for differential coexpression pattern, wherein there exists a significant difference in coexpression pattern between two phenotype conditions. These changes of coexpression patterns generally arise due to significant change in regulatory mechanism across different conditions governed by natural progression of diseases. Here we develop a novel multiobjective framework DiffCoMO, to identify differentially coexpressed modules that capture altered coexpression in gene modules across different stages of HIV-1 progression. The objectives are built to emphasize the distance between coexpression pattern of two phenotype stages. The proposed method is assessed by comparing with some state-of-the-art techniques. We show that DiffCoMO outperforms the state-of-the-art for detecting differential coexpressed modules. Moreover, we have compared the performance of all the methods using simulated data. The biological significance of the discovered modules is also investigated using GO and pathway enrichment analysis. Additionally, miRNA enrichment analysis is carried out to identify TF to miRNA and miRNA to TF connections. The gene modules discovered by DiffCoMO manifest regulation by miRNA-28, miRNA-29 and miRNA-125 families.

## Introduction

The typical course of HIV infection starts with a acute human immunodeficiency virus (HIV) infection, also known as primary HIV infection or acute retroviral syndrome^1, 2^. In this stage large amount of viruses are produced in human body and the amounts of CD4+ cells begins to drop downward^3^. Subsequently the immune response will retrieve the viral load to a certain level, called ‘viral set point’ which is a marginally stable state of HIV virus load in human body. A small proportion of HIV infected individual remain clinically stable for a long periods and have been referred as long-term nonprogressors^3^. In most of the situation the acute phase of infection progresses to the latent phase (chronic) accompanied with markedly diminishing CD4+ cell count. This results constitutional symptoms of HIV in human body that ultimately leads to AIDS.

Microarray based coexpression network analysis has been demonstrated to be an emerging field to investigate the coregulation pattern in gene regulation. This also offers an opportunity for discovering the differences in gene expression pattern across different stages of disease progression. Several studies have been proposed to reveal the changes in expression profile by introducing differential expression in different stages of HIV infection^4, 5^. In Hyrcza *et al.* ^6^ an analysis is carried out to detect differential regulation pattern of genes across different stages of early and chronic progression of HIV-1. They also demonstrated that expression of interferon stimulated genes is significantly increased across these stages. In a similar study Li *et al.* ^7^ proposed a framework to investigate stage specific expression pattern during HIV-1 infection by utilizing differential expression analysis of lymphatic tissue microarrays. In a recent study^8^ topological characteristics of coexpression modules are investigated through HIV infection stages. But all these studies are confined in investigating the extent of differences in expression patterns over different stages of HIV progression. Recently coexpression and differential coexpression analysis have been demonstrated to be powerful methods for exploring coexpression patterns and investigating intrinsic relationships between a set of coexpressed genes (or clusters) across different conditions of a specific disease. Co-expression analysis deals with the degree of coexpression under a certain condition whereas differential coexpression analysis looks for the differences in coexpression of a set of gene pairs or gene clusters across different experimental conditions^9–11^. As modules are the best representative of any network, a proper analysis of differentially coexpressed modules may reveal significant changes in coregulation pattern across different stages of disease progression.

Several studies have been developed to study the change in coexpression patterns across normal and disease states. Computational methodologies have been developed to investigate differential coexpression (DC) patterns by finding differentially coexpressed gene pairs or set of gene clusters (modules) that show a significant change in coexpression between normal and disease states^9, 12–15^. Other approaches seek to identify clusters in one condition and test whether these modules show change in coexpression patterns in a different condition. For example CoXpress^12^ utilizes hierarchical clustering to explore the relationship between genes. The gene modules are defined by cutting the tree at specific level. CoXpress then uses a resampling technique to identify those groups which are coexpressed in one set of experiment but not in other. Another approach called DiffCoex^13^ is developed to identify DC modules across two different conditions. To quantify DC, DiffCoex utilizes a statistical framework. DiffCoex transforms the DC scores into dissimilarity measures and exploits a popularly used tool WGCNA (Weighted Gene Coexpression Network Analysis)^16^ for grouping the DC modules. A recently proposed method called DICER (Differential Correlation in Expression for meta-module Recovery)^5^ aims to identify gene sets whose correlation patterns differs markedly between disease and control samples. Dicer goes beyond the convenient approaches of differentially coexpressed module detection and identifies differentially coexpressed meta-modules which basically represents a class of modules where each pair of modules shows a significant change in coexpression patterns, but retains same coexpression patterns within each module.

Most of the methods are mainly used some scoring function to capture the differential coexpression and employed some searching technique to optimize it. Here the differentially coexpressed module detection problem is modeled as a multiobjective optimization problem, optimizing different criteria simultaneously. Multiobjective modeling of a problem is useful as it finds solution by optimizing different criteria simultaneously. For example, our earlier approach^17^ was to develop a multiobjective model to detect coexpression module by optimizing two cluster validity index simultaneously. In this article, we develop DiffCoMO (**Diff**erentially **CO**expressed module detection using **M**ulti-**O**bjective algorithm) for identifying differentially coexpressed modules across two different conditions of disease progression. The coexpression patterns of human CD4+ and CD8+ T cells from HIV infected individuals at two clinical stages of HIV progression viz., acute and chronic are considered for this purpose.

DiffCoMO operates on two objective functions. The first one maximizes modulewise distance between two coexpression networks of two infection stages. The second one maximizes absolute difference of module membership value of a gene within a module across two stages. The two objectives equally contribute for obtaining the Pareto optimal solutions. For identifying similarity between two gene expression profile Pearson correlation coefficient is utilized here. The significance of the identified modules are assessed by applying some statistical test based on the extent of Differential Coexpression score (*DC*_*Score*). We compare our method with other state-of-the-art techniques like DiffCoex^13^, CLICK^18^ and CoXpress^12^, and DICER^5^. We demonstrate that our method improves upon the state-of-the-art in detecting differential coexpression patterns more accurately. For biological validation we survey the GO terms and KEGG pathway enrichment of the detected differentially coexpressed modules to identify some key functionality that play important role in progression of infection from acute to chronic stage.

## Methods

In this section we describe the proposed multiobjective framework for identifying differentially coexpressed modules across two different stages of HIV infection. The following sections describe construction of differential coexpression network and a brief description of objective functions to detect modules in this network.

### Differential coexpression of gene in two phenotypes

In gene coexpression network each gene corresponds to a node and the correlation between each pair of genes corresponds to an edge between nodes. Correlation is generally designated as a good measure that is able to capture positive and negative dependence simultaneously between a pair of variables. So, to determine similarity between two gene expression profiles Pearson correlation coefficient is generally used. So, gene coexpression network corresponds to a of similarity matrix which encodes the connection strength between two pair of genes^16^. Some adjacency functions are useful to perform soft or hard thresholding on similarity matrix for building an adjacency matrix which corresponds to unweighted or weighted network respectively. From a network perspective the coexpression network can be formally defined as: *G* = (*V*, *E*, *W*), where *V* represents nodes (genes) *E* represents edges corresponding to a pair of nodes and *W* is an weight function defined as *W* : *E* → [−1, 1], which represents the correlation between the pair of nodes.

Differential coexpression generally deals with the change in coexpression patterns across two different phenotypes. To deals with it, one needs to define a measure that approximately reflects coexpression change of a gene pair. A simple way to compute it is to calculate the absolute difference of similarity values across two phenotypes. This can be formally stated as:

1 $$D{C}_{i,j}^{p1,p2}=|Sim{({x}_{i},{x}_{j})}^{p1}-Sim{({x}_{i},{x}_{j})}^{p2}|,$$

where *p*_1_, *p*_2_ are two different phenotype conditions, and *x*_*i*_, *x*_*j*_ represent expression profile of *gene*_*i*_ and *gene*_*j*_ respectively. Here *Sim*(*x*_*i*_, *x*_*j*_)^*p*^ signifies Pearson correlation between *x*_*i*_ and *x*_*j*_ for phenotype *p*.

Following the same convention we can define differential coexpression score or *DC*_*Score* of a module by averaging the DC values between all pair of genes within it. The module-wise average *DC*_*score* can be defined as

2 $$DC\_Scor{e}_{{M}_{i}}=\frac{{\sum }_{i,j\varepsilon {M}_{i}}D{C}_{i,j}^{p1,p2}}{N},$$

where $D{C}_{i,j}^{p1,p2}$ represents differential coexpression score between gene *i* and gene *j* in module *M*_*i*_ across two phenotypes *p*1 and *p*2, and *N* represent number of genes in the module. Large value of this metric corresponds to modules having high differential coexpression.

Here, we have built two coexpression networks by considering expression profile of genes in acute and chronic stage of infection. A differential coexpression network is also constructed from these expression datasets.

### The DiffCoMO framework

DiffCoMO is totally based on the GA based multiobjective framework. It utilizes the popular Non-dominated Sorting GA (NSGA-II) for constructing the multiobjective framework. The multi-objective optimization problem can be formally defined as follows^19^: find the vector ${\bar{x}}^{\ast }={[{x}_{1}^{\ast },{x}_{2}^{\ast },\ldots ,{x}_{n}^{\ast }]}^{T}$ of the decision variables satisfying the *m* inequality constraints: ${g}_{i}(\bar{x})\ge 0,i=1,2,\ldots ,m$, and *p* equality constraints ${h}_{i}(\bar{x})=0,i=1,2,\ldots ,p$, that optimizes the vector function $\bar{f}(\bar{x})={[{f}_{1}(\bar{x}),{f}_{2}(\bar{x}),\ldots ,{f}_{k}(\bar{x})]}^{T}$. The constraints define the feasible region *F* containing all the admissible solutions. The vector ${\bar{x}}^{\ast }$ denotes an optimal solution in *F*.

The concept of *Pareto optimality* ^19^ is useful in the domain of multi-objective optimization. From the viewpoint of minimization problem the definition of Pareto optimality may be given as follows: A decision vector ${\bar{x}}^{\ast }$ is called Pareto optimal if and only if there is no $\bar{x}$ that dominates ${\bar{x}}^{\ast }$, i.e., there is no $\bar{x}$ such that $\forall i\in \{1,2,\ldots ,k\},{f}_{i}(\bar{x})\le {f}_{i}({\bar{x}}^{\ast })$ and $\exists i\in \{1,2,\ldots ,k\},{f}_{i}(\bar{x}) < {f}_{i}({\bar{x}}^{\ast })$. In other words, ${\bar{x}}^{\ast }$ is Pareto optimal if there exists no feasible vector $\bar{x}$ which causes a reduction on some criterion without a simultaneous increase in at least one another. Pareto optimum usually encompasses a set of solutions generally called *non-dominated* solutions.

For building up the chromosome structure DiffCoMO encodes a set of nodes as a chromosome. For example, a chromosome *p*_1_, *p*_2_ … *p*_*n*_ where *p*_*i*_ is an integer denoting the index of a gene (or node), represents a differentially coexpressed gene modules. The edge weights between these nodes represent differential coexpression score. Here, the population size is taken as 50 and the algorithm runs for 200 generations. All the Pareto optimal solutions obtained in the final generation are taken as resulting modules.

For starting from a reasonable position we construct the initial population as a set of modules with high DC scores. For this purpose, we first dichotomize the differential coexpression matrix by choosing a threshold of 0.5. Next, we randomly chose some substructure consisting of all 1s from this resulting binary matrix. To find out all 1s substructures we apply a biclustering technique^20^ and randomly pick up some of the biclusters consisting of all 1s. Here, union of rows and columns of each bicluster is treated as a chromosome, which comprise the initial population.

#### Representation of objective functions

To construct the objective functions we have emphasized the change of coexpression pattern of a pair of genes across the acute and chronic stages of HIV infection. Here, we have two correlation matrices which represent the expression similarity among each pair of gene expression profiles across the two infection stages.

For constructing the first objective function we have computed the distance between two correlation matrices. Let, *M*_*i*_(*pk*) represent correlation matrix of module *M*_*i*_ at stage *pk*. Consider the inner product between two matrices *M*_*i*_(*p*1) and *M*_*j*_(*p*2) of infection stages *p*1 and *p*2 which satisfies:

3 $$\langle {M}_{i}(p1),{M}_{i}(p2)\rangle =tr\{{M}_{i}(p1){M}_{i}(p2)\}\le {\Vert {M}_{i}(p1)\Vert }_{F}{\Vert {M}_{j}(p2)\Vert }_{F}.$$

For two infection stages *p*1 and *p*2 the distance metric is defined as

4 $${f}_{1}=Dis{t}_{{M}_{i},{M}_{j}}=1-\frac{tr\{{M}_{i}(p1)\}tr\{{M}_{j}(p2)\}}{{\Vert {M}_{i}(p1)\Vert }_{F}{\Vert {M}_{j}(p2)\Vert }_{F}},$$

where *tr*. represent the trace operator and ||.||_*F*_ represents Frobenius norm. The metric $Dis{t}_{{M}_{i},{M}_{j}}$ represents the distance between two correlation matrices *M*_*i*_ and *M*_*j*_. It becomes zero when the two correlation matrices are equal, and is equal to one if the matrices differ maximum amount. The distance is motivated from^21^, where the metric is used to characterize the change of spatial structure of multiple-input multiple-output (MIMO) channels.

For building the second objective we utilize module eigenegene^16^ based measure. Module Eigenegene is generally considered as a representative of the whole module. It is defined as the first left singular vector of the expression matrix corresponding to the module. Suppose *X* ^(*q*)^ represent gene expression data corresponding to the module *q*. The singular value decomposition of *X* ^(*q*)^ (*m* × *n*) provides three matrices *U*, *V* and *D* as follows: *X* ^(*q*)^ = *UDV* ^*T*^ where *U*(*u*_1_, *u*_2_, … *u*_*min*(*m*,*n*)_) and *V*(*v*_1_, *v*_2_, … *v*_*min*(*m*,*n*)_) are the matrices of left and right singular vectors and *D*(*d*_1_, *d*_2_, … *d*_*min*(*m*,*n*)_) is a diagonal matrix containing singular values. The first column of *U* ^(*q*)^ (i.e., *u*1^(*q*)^) is referred to as module eigengene. Module eigenegene generally explains the highest amount of variation in the module expression data. The Pearson correlation between expression profile of a gene and a module eigengene is designated as the module membership value of that gene. For a module, the module membership values of all genes are calculated for two infection stages. Let, $MM\_{g}_{i}^{{m}_{i}^{p}}$ represents the module membership value of a gene *g*_*i*_ of module *m*_*i*_ at infection stage *p*. For each of the module we have computed this metric for two infection stages *p*1 and *p*2. Particularly, for each gene in a module we have computed the following metric:

5 $$diff\_MM\_{{g}_{i}}^{p1,p2}=|MM\_{g}_{i}^{{m}_{i}^{p1}}-MM\_{g}_{i}^{{m}_{i}^{p2}}|$$

This signifies the absolute difference between module membership value of a gene in two different infection stages. We take the average of all these values inside a module. Thus our second objective function is defined as

6 $${f}_{2}=\frac{{\sum }_{{M}_{i}\in M}{\sum }_{{g}_{i}\in {M}_{i}}diff\_MM\_{g}_{i}^{p1,p2}}{N},$$

where, *M* is the module set, and *N* represent number of modules.

### Testing of objective functions

**Figure 1:**
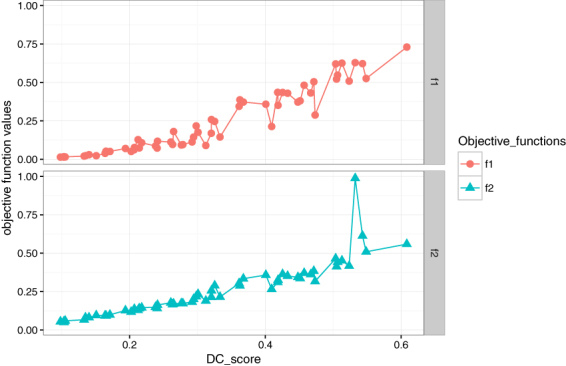
Figure shows the plot of *DC*_*Score* vs objective function values. Values of both objective functions increase with the *DC*_*Score* values.

To test the objective functions are capable for detecting the differential coexpression pattern we have performed an analysis. For each of the metric we have investigated the distribution of *DC*_*Score* which are believed to be higher for differential coexpression modules. Figure 1 shows the distribution of objective function values with *DC*_*Score*. Here we see that there exist a strong correlation between *f*1(or *f*2) with *DC*_*Score*. The way we define the first objective *f*1 is very straightforward. For a module, it compute the distance between two correlation matrices which comes from two different infection stages. Note that *f*1 actually mimics the value of *DC*_*Score* in a different way. Figure 1 also proves the same statement. This is also true for the other objective function *f*2. Module membership of a gene in a module characterizes the overall expression similarity with the other genes within the module. For a gene, if this value differ significantly across two infection stages, it actually reflects the change of expression similarity with the other genes within the module. The increasing value of *f*2 with *DC*_*Score* also proves this statement.

## Results

In this section we describe the performance of our proposed method with that of some state-of-the-art technique like DiffCoEx, CoXpress and CLICK.

### Description of Dataset

**Figure 2:**
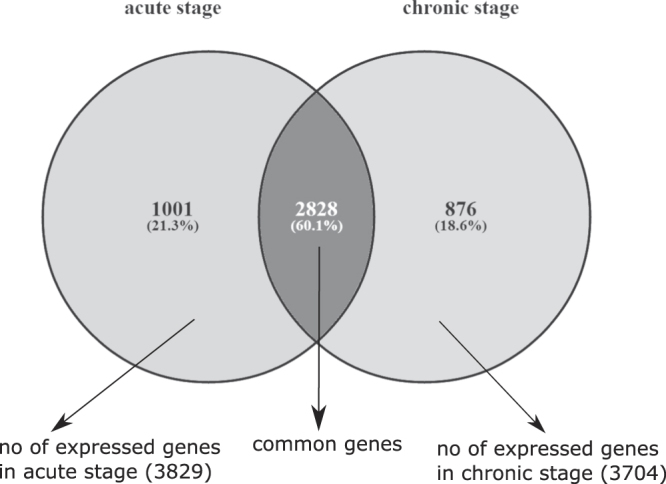
Venn diagram showing the overlaps of expressed genes in acute and chornic stages. 2828 genes are common between 3829 expressed genes of acute stage and 3704 expressed genes of chronic stage.

We downloaded the series GSE6740 dataset from GEO database (http://www.ncbi.nlm.nih.gov/geo/) for HIV-1 expression data. The dataset consist stage specific gene expression of human CD4+ and CD8+ T cells. It is prepared by examining the gene expression profiles in *ex vivo* human CD4+ and CD8+ T cells from untreated HIV-infected individuals at different clinical stages of disease progression^6^. The dataset consists of 22284 genes with 10 samples in each stage. The platform used for the analysis is Affymetrix Human Genome U133A Array. Some genes are represented by multiple probe sets. Also the other probe sets are not fully annotated, so for consistency we refer to probe sets as “genes” throughout the article. A gene is considered expressed if it is called as “present” or “marginal” in all of the samples in the given dataset. This is determined by “mas5calls” function (Bioconductor “affy” package) in R. It performs the Wilcoxon signed rank-based gene expression presence/absence detection call to determine whether the transcript of a gene is detected (present) or undetected (absent). This results 3829 and 3704 expressed genes in acute and chronic stage, respectively. We select 2828 common genes among the expressed genes for our analysis. The scenario is shown in Fig. 2.

**Figure 3:**
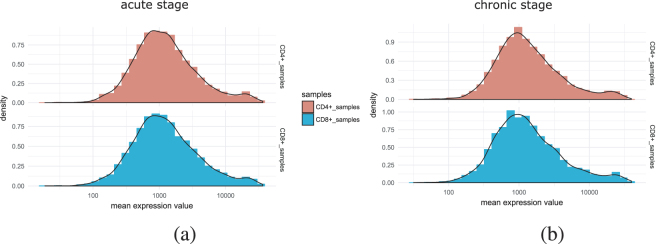
Figure shows the distribution of mean expression values of all expressed genes in CD4+ and CD8+ samples in acute and chronic stages.

**Figure 4:**
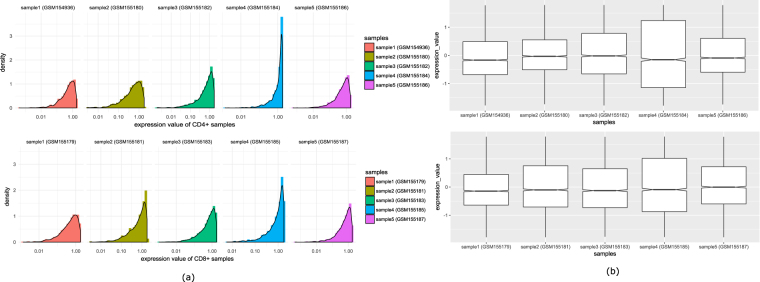
Comparison of expression values of CD4+ and CD8+ samples in acute stage. Panel (a) shows the distribution of five samples of CD4+ cell (GSM154936, GSM155180, GSM155182, GSM155184 and GSM155186) and five sample of CD8+ cell (GSM155179, GSM155181, GSM155183, GSM155185 and GSM155187). Panel (b) shows the boxplots of the corresponding samples.

**Figure 5:**
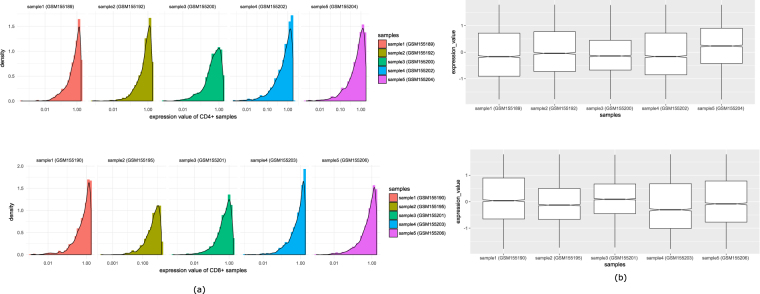
Comparison of expression values of CD4+ and CD8+ samples in chronic stage. Panel (a) shows the distribution of five samples of CD4+ cell (GSM155189, GSM155192, GSM155200, GSM155202 and GSM155204) and five sample of CD8+ cell (GSM155190, GSM155195, GSM155201, GSM155203 and GSM155206). Panel (b) shows the boxplots of the corresponding samples.

It can be noted that each stage consists of ten samples, five samples are for CD4+ T cells and five samples are for CD8+ T cells. To know whether there is any difference in the expression patterns of CD4+ and CD8+ T cells in each stage, we perform an analysis. First, we separate the CD4+ and CD8+ samples of acute and chronic stage. For each stage, we plot the distribution of mean expression values of all expressed genes in CD4+ and CD8+ samples. Figure 3 (panel-(a) and panel(b)) shows the distribution. Moreover, we investigate the distribution of all CD4+ and CD8+ samples. Figures 4 and 5 show the density plot and box plots of all the samples for acute stage and chronic stage respectively. It appears from the figures that there is no such distinguishable change of expression patterns between CD4+ and CD8+ samples in acute and chronic stages.

### Comparing DiffCoMO with some state-of-the-art

Here, we compare the performance of our proposed method with that of some other method like CLICK, DiffCoex and CoXpress. To assess the performance of all the methods and conduct a fair comparison we compare the ability of the methods to detect Differential coexpression pattern within each modules.

**Figure 6:**
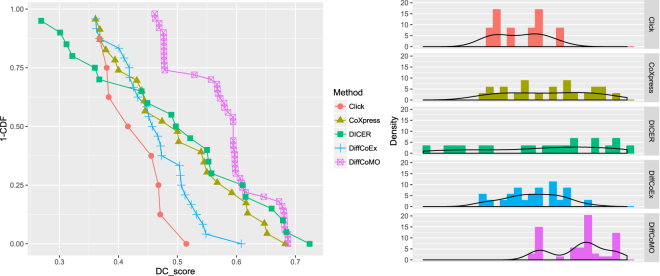
Distribution of *DC*_*Score* - the left pane show the fraction of identified modules having *DC_Score* score above 0.35, while the right pane show the distribution of *DC*_*Score* for DiffCoex, CoXpress, CLICK and the proposed DiffCoMO algorithm.

We applied CLICK, CoXpress, DiffCoex and proposed method DiffCoMO on the acute and chronic stage of HIV-1 expression dataset and find set of differentially coexpressed modules in each case. We inspect absolute change in correlation of each detected modules. For this purpose we calculate *DC*_*Score* of identified modules of all the methods and plot these in Fig. 6(a,b). The left panel of the figure shows the distribution of *DC*_*Score* with value 0.35 or above with the fraction of identified modules for DiffCoex, CoXpress, CLICK, DICER and the proposed method DiffCoMO. The right panel shows the distribution of *DC*_*Score* for the five state-of-the-arts.

From the Fig. 6 panel (a) it is clear that DiffCoMO has higher proportion of modules having better *DC*_*Score*. Moreover, from Fig. 6 panel (b) it can be also noticed that all the modules in DiffCoMO have *DC*_*Score* comparatively better than the other competing methods.

**Figure 7:**
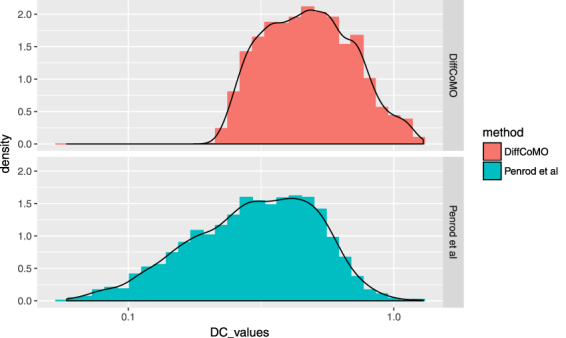
The upper pane of the figure shows the distribution of *DC*_*values* of all the gene pairs belonging to the differentially coexpressed modules obtained from DiffCoMO. The lower pane shows the same for the method proposed in ref. 22.

The metric *DC*_*Score* represents average DC_values between all pair of genes within a module. To know the extent of differential coexpression between all pair of genes within each module we plot the distribution of DC_values of all the genes. Here, we have compared the proposed technique with the method proposed in ref. 22. By following the same methodology proposed in ref. 22 we compute differentially coexpressed gene pairs across acute and chronic stage of infection. We plot the distribution of DC_values of all the gene pairs obtained from the analysis and compared this with DiffCoMO. Figure 7 shows the distributions. It is evident form the figure that DiffCoMO also has the capability to detect differentially coexpressed gene pairs across two infection stages of disease progression.

### Identified modules are statistical significant

**Figure 8:**
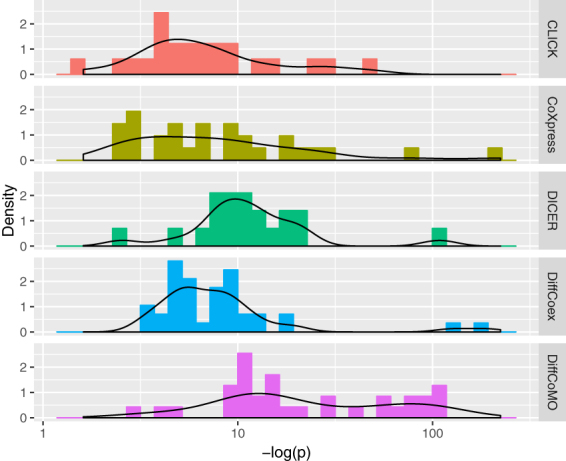
Figure shows the distribution of *p*-*values* obtained from Jennrich test, for DiffCoex, CoXpress, CLICK and the proposed DiffCoMO algorithm. For better visualization we plot the distribution of −*log*(*p*) values obtained from different methods.

To investigate whether the difference in correlation pattern in identified modules is statistically significant, we have performed a statistical test. For this, we have used the Jennrich test^23^ which compares equality of two matrices. It identifies the differences between two correlation matrices to the averages of these two using a chi square test. Using this test we have compared two correlation matrices corresponding to acute and chronic stage, of differential coexpression modules. The distribution of p-values for different algorithms are shown in Fig. 8. It appears from the figure that the DiffCoMO modules show lower p-values (higher −log(p)) than the other state-of-the-art. Almost all the modules of DiffCoMO have p-value less than 0.01 which proves that the difference in correlation pattern is identified effectively.

### Measuring performance in a simulated dataset

**Figure 9:**
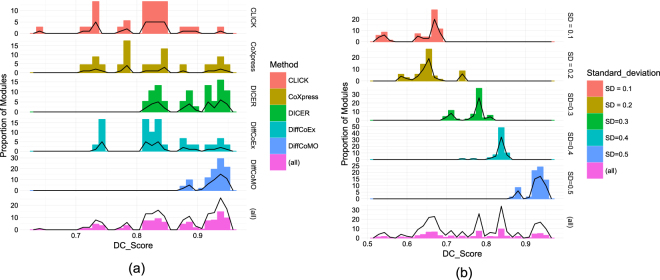
Panel (a) shows distributions of *DC*_*Score* of the identified modules for different algorithms at SD value 0.5. Panel (b) shows the distribution *DC*_*Score* of DiffCoMO modules at different SD values.

We use simulated data to evaluate the performance and investigate the correctness of our algorithm to capture the differential coexpression modules. For this purpose, we have created 5 sets of different coexpression networks from the original set, where each set contains two networks corresponding to two different stages. The networks are built by adding random values generating from normal distribution (mean (*μ* = 0)) with different standard deviation (SD) values with the original network. For a set, two different networks are cerated with a fixed SD value. In a set, to create the difference between the two generated coexpression networks, most of the cases we keep opposite sign of random numbers across the two networks. Thus we have generated 5 different sets of these simulated networks by varying the SD value from 0.1 to 0.5. Thus, the networks in a set, surely have strong difference in their correlation patterns. As the SD increases, larger random values are added to the correlation matrices, as a result the difference of the two correlation matrices are subsequently increases. So, for each SD value we have a pair of correlation matrices with weights corresponding to the random values generated by the procedure described above. Table 1 shows the average *DC*_*Score* at different SD values of all modules extracted from the simulated data by using the five different algorithms. It can be noticed from the table that the average *DC*_*Score* of all modules corresponding to each network gradually increase with higher value of SDs. This is obvious, as with the increment of SD values, the weights of both the network which generally represent correlation value is also increased, thus average *DC_Score* of all predicted modules moderately increased. For better visualization we have plot the distribution of *DC*_*Score* for each method at different SD values. Figure 9(a) shows the distributions for SD value 0.5. The distributions for other SD values are keep in Supplementary Figure. Figure 9(b) also shown the *DC*_*Score* distributions for DiffCoMO at different SD values. From the Fig. 9(b) it is clear that our proposed method DiffCoMO behave correctly with respect to the simulated data. Figure 9(a) shows DiffCOMO has the ability to detect differential coexpression pattern better than the other methods in simulated data.

**Table 1:** Performance of DiffCoMO nad DiffCoEx with respect to simulated dataset: average *DC*_*Score* of all modules identified from simulated differentially coexpressed network with increasing SD value.

	SD				
	0.1	0.2	0.3	0.4	0.5
DiffCoex	0.5296	0.6060	0.6960	0.7543	0.83503
CLICK	0.4385	0.4762	0.6608	0.7174	0.8086
CoXpress	0.4439	0.5855	0.6795	0.7294	0.8238
DICER	0.5682	0.6029	0.7149	0.7851	0.8868
DiffCoMO	0.6369	0.6517	0.7653	0.8333	0.9258

We have also investigate the performance of DiffCoex in the same simulated data. The average *DC*_*Score* of all extracted modules are listed in the Table 1.

### Biological validation of modules

In this section we biologically validate the differential coexpresed modules identified by the proposed DiffCoMO algorithm. For this purpose we have performed a Gene Ontology and pathway based analysis of the identified modules. Moreover, as coexpression results from coregulation among the genes, so change in coexpression may effect the regulation pattern also. So, we have performed an analysis to identify transcrption factors (TF) that are targets of some miRNA families. As the microRNA families are generaly known to be associated with some specific disease so we have also performed an analysis to identify the disease association with selected miRNA families associated with different transcription factors which in tern involved in some of the identified modules.

#### GO and Pathway enrichment

We have investigated what extent of Gene Ontology terms and pathways are associated with the identified differential coexpression modules. For this purpose we collected the GO terms from GO database and able to associate these terms with identified modules. From KEGG database, we also identified significant pathways that are involved in different differential coexpression modules. Table 2 shows significant GO-terms and pathways discovered from the identified modules. A careful observation on Table 2 reveals that some of the identified GO terms are common among the modules. This is because of the overlap in the identified modules. To identify the overlaps we compute a overlap score between each pair of identified modules. Overlap score between a pair of modules is defined as the number of common human proteins divided by the total number of unique human proteins in these modules. In Supplementary Table 1 we have shown the overlap scores among the identified differentially coexpressed modules.

**Table 2:** GO-terms and KEGG pathway of some identified differentially coexpressed modules.

module(Sl No.)	#genes	*DC*_*Score*	GO term(bp)	KEGG pathway
1	46	0.8216	positive regulation of molecular function (GO:0044093) (1.7E-5)	Alzheimer’s disease (1.0E-3)
2	93	0.8168	generation of precursor metabolites and energy (GO:0006091)(1.3E-5)	Alzheimer’s disease (1.1E-5)
3	102	0.8131	generation of precursor metabolites and energy (GO:0006091)(9.1E-6)	Parkinson’s disease (3.4E-3)
4	114	0.8070	positive regulation of ubiquitin-protein ligase activity during mitotic cell cycle(GO:0051437)(8.1E-5)	Alzheimer’s disease (6.6E-6)
5	126	0.8093	lymphocyte differentiation (GO:0030098) (1.5E-2)	Thyroid cancer (1.6E-2)
6	156	0.7302	anaphase-promoting complex-dependent proteasomal ubiquitin-dependent protein catabolic process(GO:0031145)(5.4E-8)	Parkinson’s disease(4.5E-6)
7	143	0.7260	DNA unwinding during replication(GO:0006268) (1.7E-4)	Intestinal immune network for IgA production (1.0E-3)
8	120	0.7100	mRNA metabolic process (GO:0016071) (2.2E-5)	Systemic lupus erythematosus (4.2E-2)
9	106	0.7003	anaphase-promoting complex-dependent proteasomal ubiquitin-dependent protein catabolic process (GO:0031145) (4.7E-6)	Proteasome (1.4E-5)

In Table 2 we show only the relevant GO-terms and pathways of those modules which have *DC*_*Score* greater than 0.7. From Table 2 it is noticeable that the pathways of Alzheimer’s disease and Parkinson’s disease are often associated with different differential coexpression modules. In ref. 24 it is demonstrated that HIV-infected peripheral blood mononuclear cells (PBMCs) show overrepresentation of neurodegenerative pathways (Alzheimer’s, Parkinson’s, ALS, Huntington’s and Prion Disease, etc). Here it is also suggested that this overrepresentation together with genome wide mapping of host gene expression may act as an indicator of possible neurological deterioration in HIV patients.

The modules are also enriched with gene sets that are belonging to different disease and disorder associated pathways like Systemic lupus erythematosus (an autoimmune disease) and Intestinal immune network for IgA production. Systemic lupus erythematosus (SLE) is a prototypic autoimmune disease caused by the malfunctioning of immune system which mistakenly attacks healthy tissue. Although SLE is rarely associated with HIV infection^25^, but earlier in ref. 26 two cases are reported where HIV infection causes SLE. Intestinal immune network for IgA production which is responsible for defense against microorganism by generating noninflammatory immunoglobulin A (IgA) antibodies, is also demonstrated to be associated with HIV infection^27^. One module is associate with the pathway Thyroid cancer. Abnormal thyroid function test results are commonly reported in HIV infection individuals^28^. In ref. 29 a case is reported where HIV infection cause thyroid medullary carcinoma.

Moreover, the modules are enriched with different significant GO-terms. For example module 1, 2, 8, and 9 are involved with similar type of GO terms. The genes in those modules are primarily involved with the activation or increase of some biological activity occurring at some molecular level like increase of enzyme activity, increases the rate of ubiquitin ligase activity or increases the rate of catalysis or binding.

**Figure 10:**
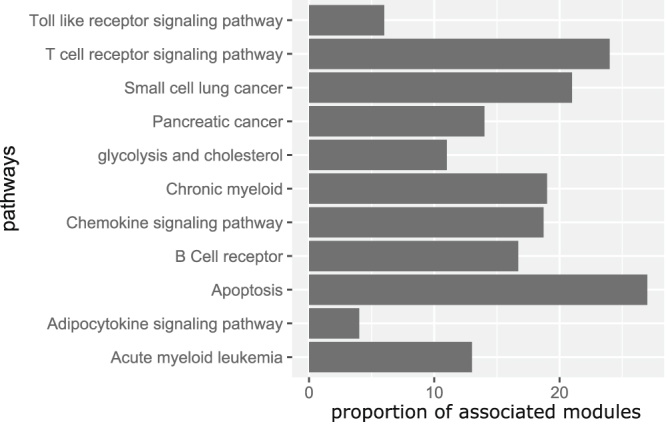
Bar diagram showing the proportion of modules associated with 11 HIV specific pathways.

To know whether the identified modules are associated with HIV specific important pathways we have performed an analysis. For this purpose, we have collected 11 HIV specific top ranked pathways form Chen *et al.* ^30^. Next, we have calculated proportion of identified modules that are associated with the pathways. We called a module is associated with a pathway if at least one gene of this pathway is belonging to this module. Figure 10 shows a bar diagram showing the association of modules with the pathways. It can be seen from the figure that some pathways like ‘Apoptosis’ and ‘T cell receptor signaling’ are associated with more number of identified modules than the other.

Hence, it is evident that the identified differentially coexpressed modules are significant and biologically meaningful.

**Figure 11:**
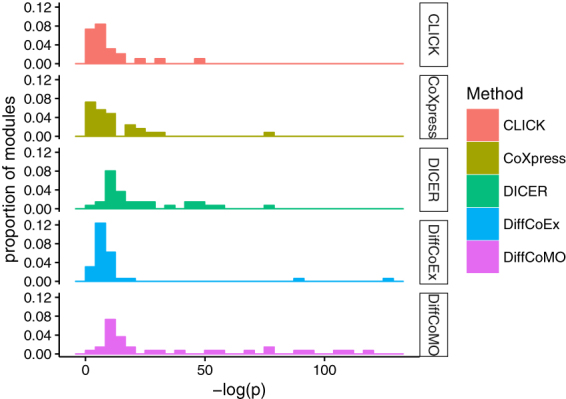
Bar diagram showing the distribution of modules at different p-values. p-value of a module closer to zero signifies that it is less likely to observe the annotation of a particular GO term to a group of genes occurs by chance. X-axis represents −*log*(*p*) whereas Y-axis represents proportion of modules identified by four algorithms. When p-value is lower −*log*(*p*) approaches to higher value.

To test whether the genes in identified modules are belonging to the same functional group we collected the p-value of individual modules identified in each of the methods. The p-value of a gene module signify the probability of observing at least *x* number of genes out of total *n* genes in the module annotated to a particular GO terms, given that the proportion of genes in the whole genome are annotated with that GO terms. The p-value is computed by comparing the GO terms shared by the genes in the module to the background distribution of annotation. So a p-value of a module closer to zero signifies that it is less likely to observe the annotation of a particular GO term to a group of genes occurs by chance. In Fig. 11 we plot a bar diagram which shows the distribution of modules at different p-values. From Fig. 11 it is depicted that large proportion of modules produced by other algorithms have higher p-values in comparison with DiffCoMO in which a significant number of modules tend to have smaller p-values (i.e. larger −*log*(*p*)). This establishes that modules identified by DiffCoMO share common biological functions and more biologically significant than the modules discovered by other algorithms.

#### miRNA enrichment

In general, change in gene coexpression patterns has strong correlation with coregulation of gene expression. So, differential coexpression of a pair of gene may be an effect of changing regulation patterns of some transcription factors. Moreover, miRNAs have an impact on HIV replication by affecting the expression of host genes which are essential in the replication process^31^. It may possible that TFs bind some miRNAs which in tern affect the expression level of genes that are required for viral replication. So, it is essential to investigate the association between TF to miRNA, miRNA to TF regulation and miRNA to gene regulation.

**Figure 12:**
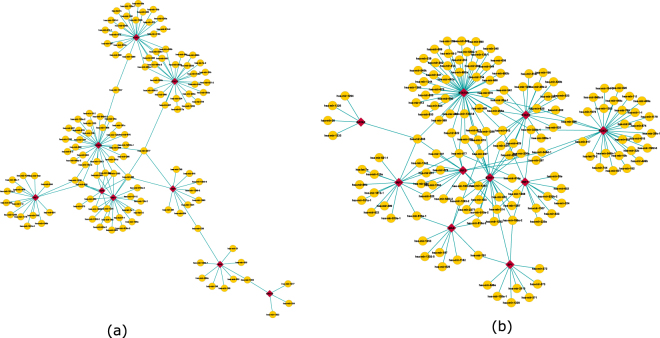
Tf-miRNA network. Here the red triangle shape nodes represent TFs identified from the differential coexpressed modules. The pink circle represent miRNAs regulated by those TFs in their upstream region (USR) (shown in panel (a)) and in downstream region (DSR) (shown in panel (b)).

**Figure 13:**
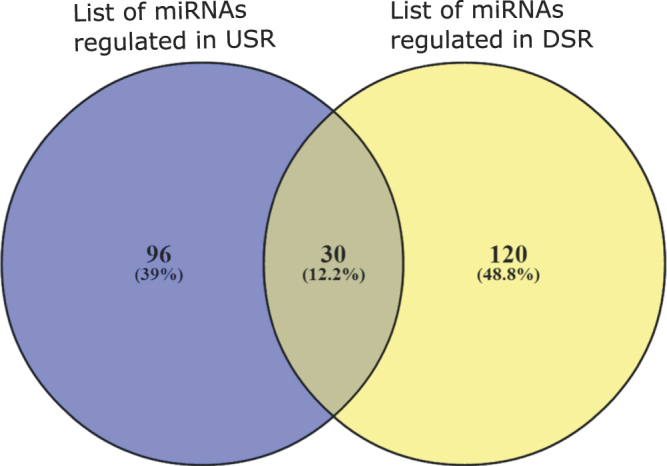
Venn diagram showing the overlaps of two miRNA list.

To investigate TF to miRNA connections we utilize a putative TF-miRNA repository PuTmir^32^. PuTmir stores the information about the regulatory activity of miRNAs in their upstream region (USR) as well as downstream region (DSR). We choose to use PuTmir because it keeps the information about TFs which binds to the DSR of miRNAs. Apart from PuTmir we have also searched existing literatures^33, 34^ to find more TFs that show altered expression in HIV infection. We found total 12 TFs which are associated with the identified modules. We look through the PuTmir database to identify the regulated miRNAs in which the identified TFs bind. We collect the miRNA list for the corresponding TFs which binds to the DSR and USR, separately. To get a better visualization of the identified TFs and associated miRNAs we create two networks between TFs and regulated miRNAs based on the two binding regions (USR and DSR) of miRNAs. Figure 12 shows these two bipartite network. From this figure we see that the identified TFs bind 126 miRNAs in the USR and 150 miRNAs in DSR. Here, the red diamond shape nodes represent TFs and yellow color nodes represent the associated miRNAs. The Venn diagram in Fig. 13 shows that 20 miRNAs are common between the two list of miRNAs. Thus, TFs binds in both USR and in DSR of these 20 miRNAs. The associated miRNAs also includes miR-29-b, miR-29-a, miR-28 and miR-125 which are demonstrated to play an important role HIV replications^31^. It^35^ it is established that human miR-29-a and miR-29-b are expressed in Peripheral Blood Mononuclear Cells (PBMC). These miRNAs are responsible for downregulating the expression of HIV-1 protein Nef^35^. In ref. 36 it is also demonstrated that miR-28 and miR-125 which are found in resting primary CD4+ T cells target 3′ ends of HIV-1 messenger RNAs, thus inhibits HIV-1 production in resting primary CD4+ T cells.

For investigating the miRNA to TFs connections we have used a experimentally validated miRNA-target interaction database miRTarBase^37^. We have found 20 TFs in our identified modules, which are regulated by miRNAs. Some non-TF genes in the modules are also found to be regulated by the same set of miRNAs. We have listed all the miRNA-TF and miRNA-non-TF interactions in Supplementary Table 2 and Supplementary Table 3. It is noticed from the tables that the identified modules are enriched with differentially coexpressed genes that are targeted by some miRNAs like miRNA-28, miRNA-29, miRNA-125 families. It is established that miR-29 families are responsible for suppression of HIV infection^31^. It is also verified that miRNA-28 and miRNA-125 families target the 3′ UTR of HIV-1 transcripts that shifts HIV infection to latency stage^38^.

To investigate if the identified TFs have more miRNA connections than expected by chance we performed a statistical test. For this, we have collected 300 set of random modules retaining the size of each module same as original. We count number of connections between miRNAs and regulated TFs. As we are not aware of the distribution of these connections so nonparametric test is the best option here. We have utilized Wilcoxon Ranksum test here. The resulting p-value (8.6733e-10) is significantly low which indicates that the identified TFs in original module have more miRNA connections than expected by chance.

### Performance of DiffCoMO in expression data with large number of samples

**Figure 14:**
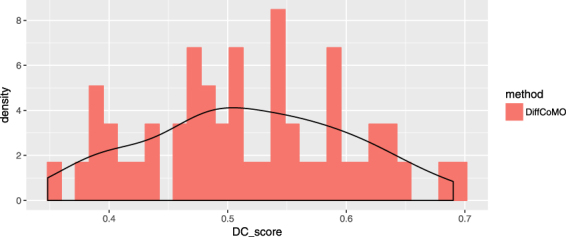
Distribution of *DC*_*Score* of all differentally coexpressed modules identified by DiffCoMO in dataset GSE18842.

In this section, we have studied the performance of DiffCoMO in microarray gene expression data having sufficiently large number of samples. Here, we have downloaded the series GSE18842 dataset from GEO database (http://www.ncbi.nlm.nih.gov/geo/) which consists 91 non-small cell lung cancer (NSCLC) samples, among them 46 samples are tumors and 45 samples are controls. We have pre-processed the data by following the same procedure mentioned in section. Finally, we have selected 3527 expressed genes for our analysis. We have applied DiffCoMO to detect differential coexpressed module in this data. We calculate the *DC*_*Score* value of all identified modules and plot this in Fig. 14. Figure shows most of the identified modules have *DC*_*Score* higher than 0.5.

## Conclusion

In this study we have developed a multiobjective framework DiffCoMO to identify differential coexpression modules from two microarray dataset corresponding to two different phenotypes. DiffCoMO operates with two objective functions. The first one maximizes the distance between two correlation matrices constructed from two different infection stages. Second objective function is built using eigengene based measure. It maximizes the difference of module membership value of gene across two infection stages. We compared the performance of DiffCoMO with four other state-of-the-art algorithms: CoXpress, DiffCoEx, Click and DICER. DiffCoMO performs better than other methods for capturing the differential coexpression patterns.

We have also measured the performance of DiffCoMO algorithm with respect to simulated dataset. The simulated study validate the correctness of our algorithm to capture the differential coexpression patterns. As random values of opposite sign (mean = 0, standard deviation (SD) = 0.1 to 0.5) are added to the correlation values of two matrices, the resulting differential coexpression matrix contains high values. So, the mean *DC*_*Score* of identified modules in these simulated data are increasing with SD values ranging from 0.1 to 0.5, as expected. DiffCoMO shows a strong increasing pattern of mean *DC*_*Score* with increasing SD values.

Differential correlation often caused by change in corregulation patterns of a set of genes by a common regulator or TF. So, we performed an analysis to find the corregulation patterns of identified TFs from the extracted modules. The regulation patterns of miRNAs that are regulated by those TFs are also investigated to see whether these miRNAs are associated with some specific disease. All the identified miRNAs are associated with different cancer associated disease. Thus we can conclude that DiffCoMO can be used to pick out disease specific miRNA families. The identified TFs also have significantly high miRNA connection than expected by chance.

In most of the situation the acute phase of HIV-1 infection progresses to the latent phase (chronic) accompanied with markedly diminishing CD4+ cell count. This results constitutional symptoms of HIV in human body that ultimately leads to AIDS. But A small proportion of HIV infected individual remain clinically stable for a long periods and have been referred as long-term nonprogressors. So, it is important to know the extent of differential coexpression changes of modules across acute to chronic stages along with acute to nonprogressor stage. As in nonprogressor stage HIV infected individual remain clinically stable for a long periods so it is important to know the change in regulation pattern of TFs among the three stages of progression viz., acute, chronic and non-progressor. We are now working in this direction.

## Electronic supplementary material

Supplementary_figure1

Supplementary_table1

Supplementary_table2

supplementary_table3
