# Supplementary material for: Identifying differentially coexpressed module during HIV disease progression: A multiobjective approach
Source: Sci Rep. 2017 Mar 7;7:86. doi: 10.1038/s41598-017-00090-2 (PMC5428367; doi:10.1038/s41598-017-00090-2)
Supplement: Supplementary file 1 — Supplementary_figure1 [file 41598_2017_90_MOESM1_ESM.pdf]

Identifying differentially coexpressed module during HIV disease progression: A multiobjective approach.

Sumanta Ray[1,*] **and Ujjwal Maulik**[2]
[1]Department of Computer Science and Engineering, Aliah University, Kolkata-700156, India
[2]Department of Computer Science and Engineering, Jadavpur University, Kolkata-700108, India
[*]sumantababai86@gmail.com

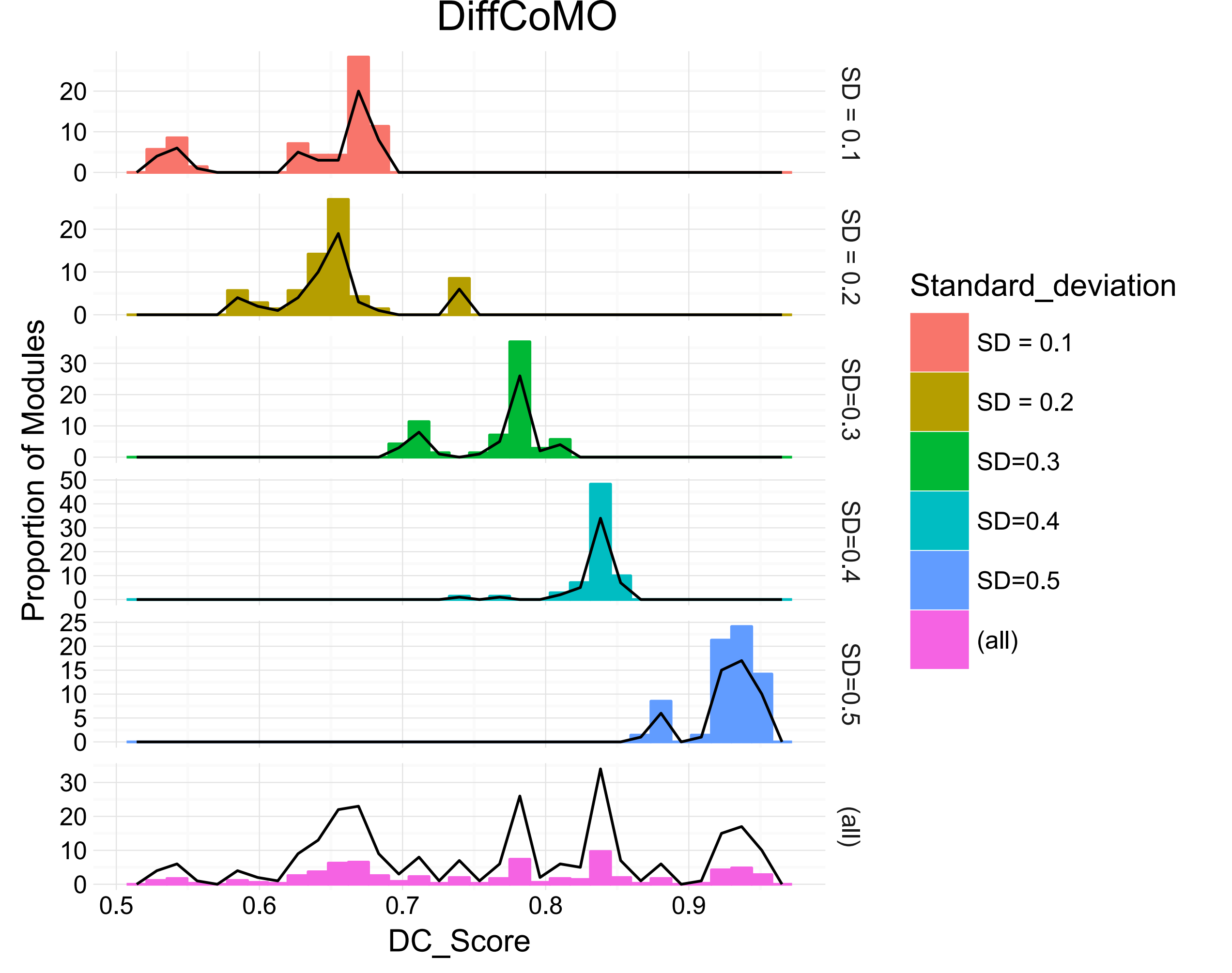

DiffCoMO
Proportion of Modules
DC_Score
0.5
0.6
0.7
0.8
0.9
SD = 0.1
SD = 0.2
SD=0.3
SD=0.4
SD=0.5
(all)
Standard_deviation
SD = 0.1
SD = 0.2
SD=0.3
SD=0.4
SD=0.5
(all)

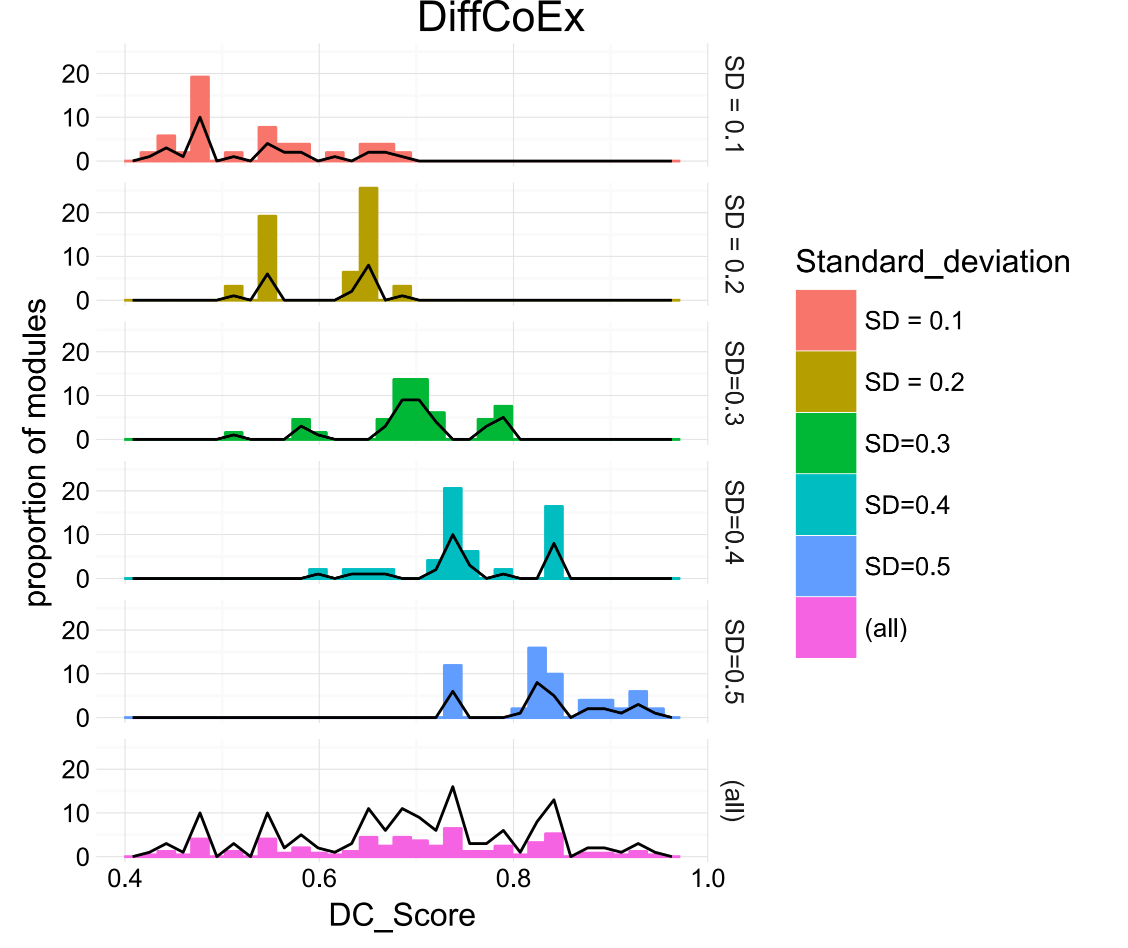
DiffCoEx
proportion of modules
DC_Score
SD = 0.1
SD = 0.2
SD=0.3
SD=0.4
SD=0.5
(all)
Standard_deviation
0
10
20
0.4
0.6
0.8
1.0

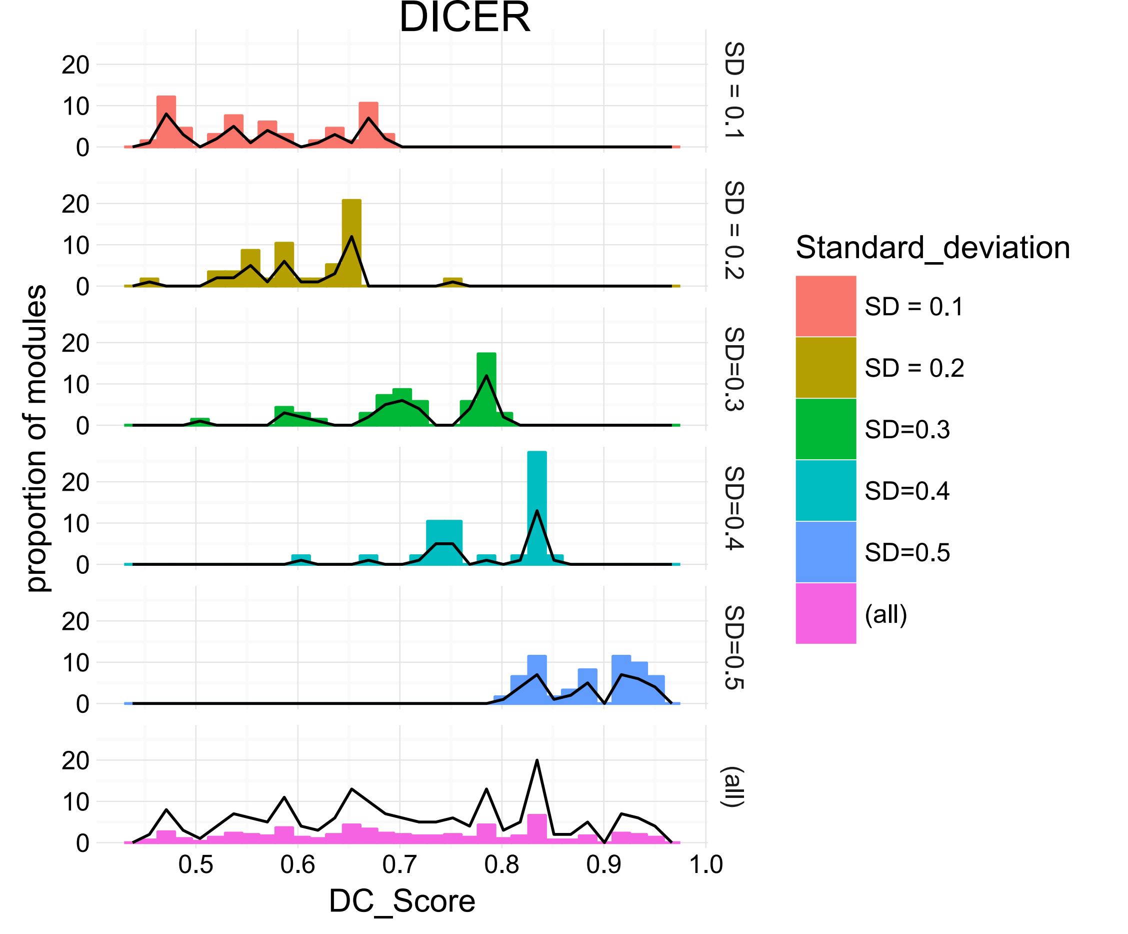

DICER
proportion of modules
DC_Score
0.5
0.6
0.7
0.8
0.9
1.0
0
10
20
SD = 0.1
SD = 0.2
SD=0.3
SD=0.4
SD=0.5
(all)
Standard_deviation
SD = 0.1
SD = 0.2
SD=0.3
SD=0.4
SD=0.5
(all)

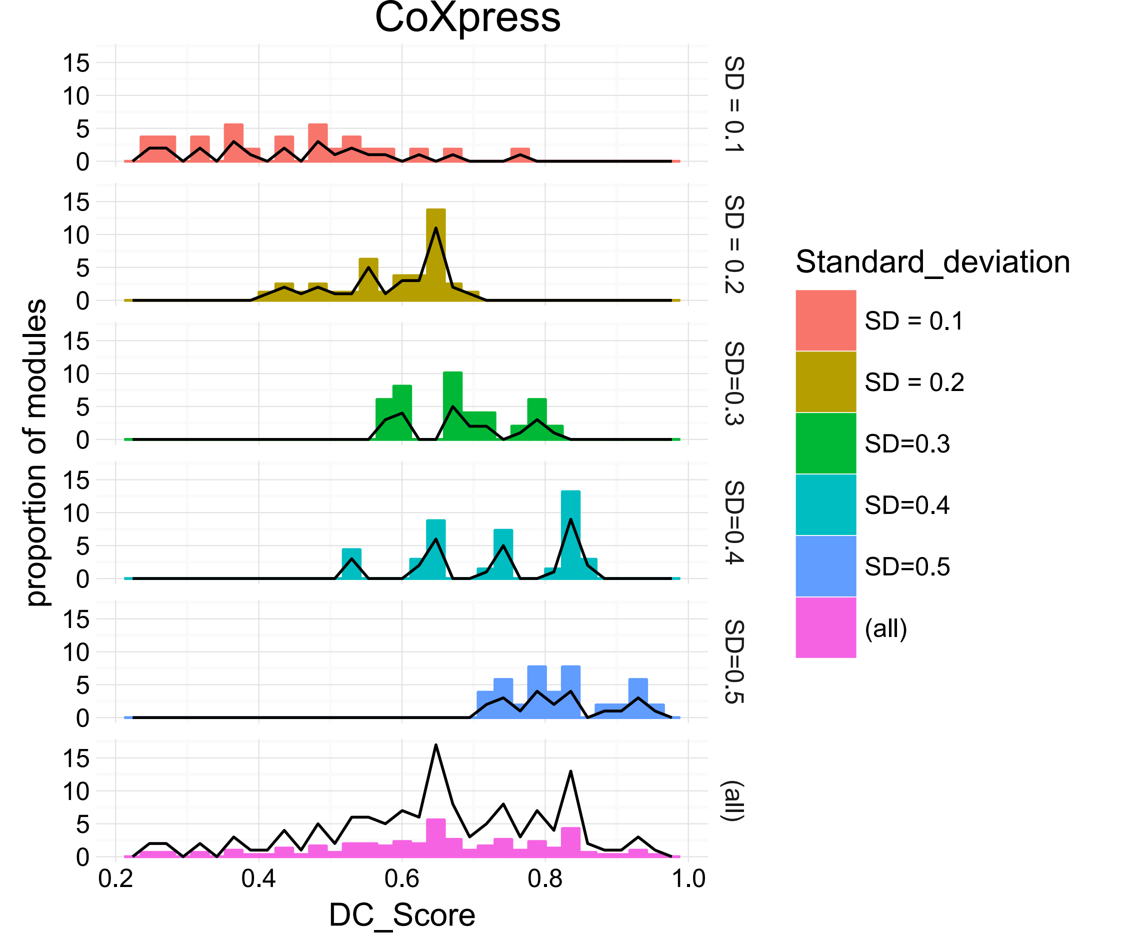

CoXpress
proportion of modules
DC_Score
0.2
0.4
0.6
0.8
1.0
0
5
10
15
SD = 0.1
SD = 0.2
SD=0.3
SD=0.4
SD=0.5
(all)
Standard_deviation
SD = 0.1
SD = 0.2
SD=0.3
SD=0.4
SD=0.5
(all)

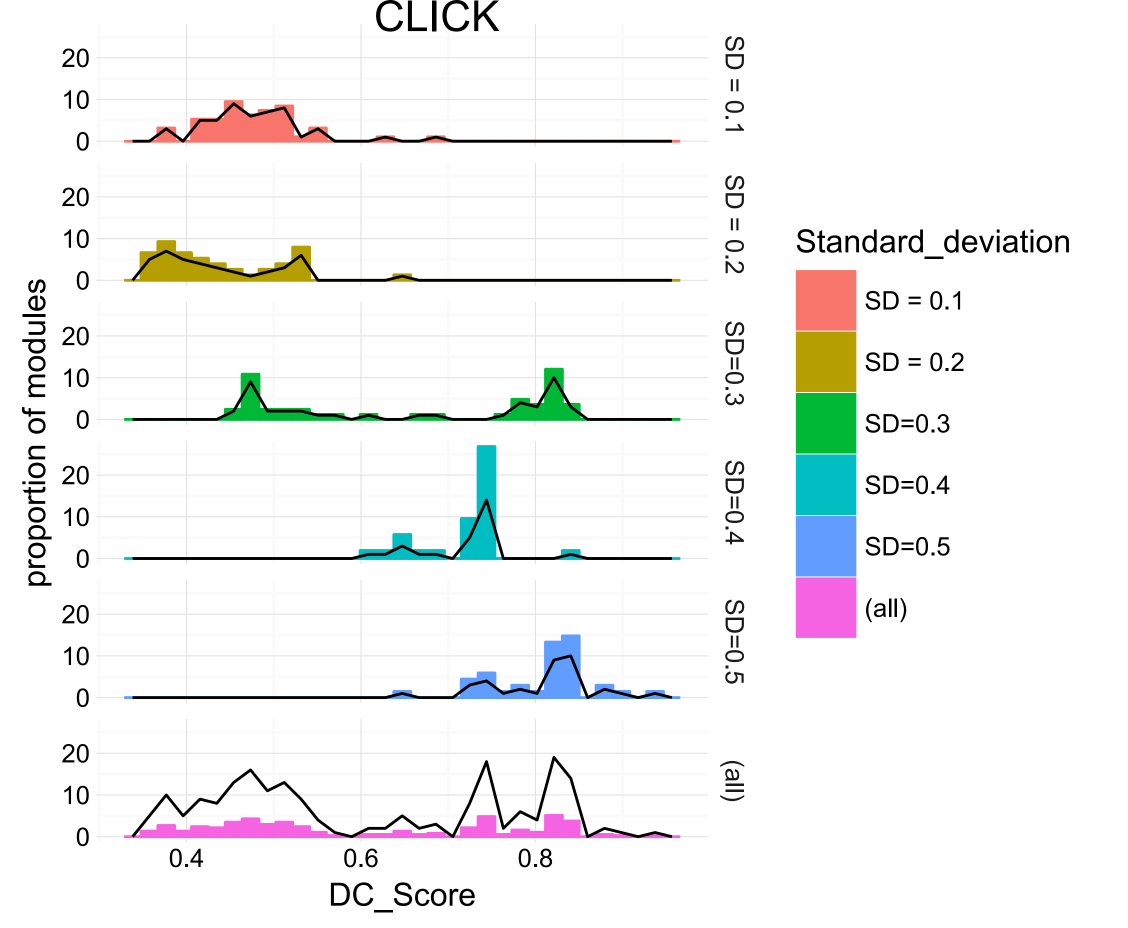
CLICK
proportion of modules
DC_Score
0.4
0.6
0.8
0
10
20
SD = 0.1
SD = 0.2
SD=0.3
SD=0.4
SD=0.5
(all)
Standard_deviation
SD = 0.1
SD = 0.2
SD=0.3
SD=0.4
SD=0.5
(all)
