# Supplementary material for: Identifying differentially coexpressed module during HIV disease progression: A multiobjective approach
Source: Sci Rep. 2017 Mar 7;7:86. doi: 10.1038/s41598-017-00090-2 (PMC5428367; doi:10.1038/s41598-017-00090-2)
Supplement: Supplementary file 2 — Supplementary_table1 [file 41598_2017_90_MOESM2_ESM.pdf]

# Identifying differentially coexpressed module during HIV disease progression: A multiobjective approach.

**Sumanta Ray**[1,*] **and Ujjwal Maulik**[2]
[1]Department of Computer Science and Engineering, Aliah University, Kolkata-700156, India
[2]Department of Computer Science and Engineering, Jadavpur University, Kolkata-700108, India
[*]sumantababai86@gmail.com

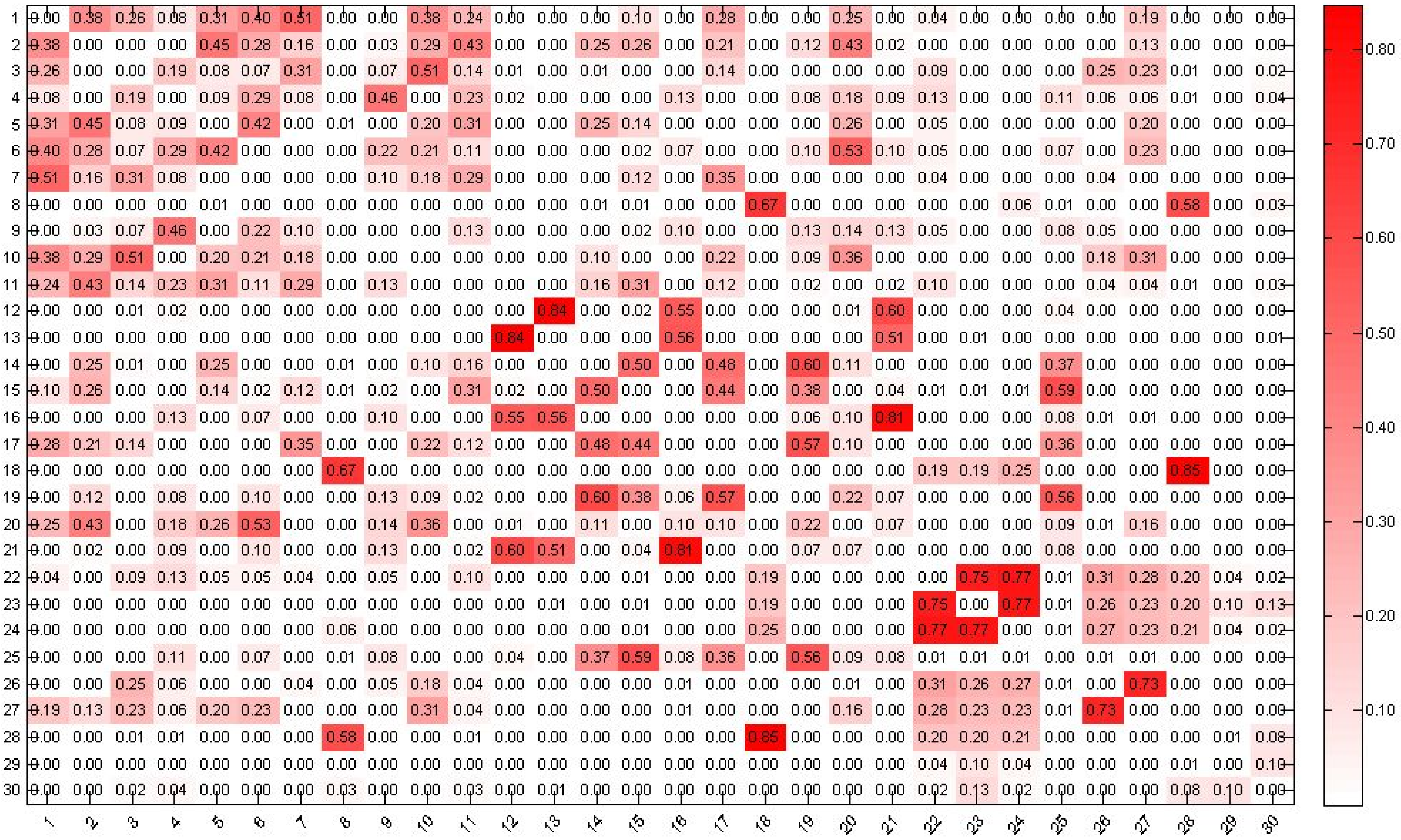

1 0.00 0.38 0.26 0.08 0.31 0.40 0.51 0.00 0.00 0.38 0.24 0.00 0.00 0.00 0.10 0.00 0.28 0.00 0.00 0.25 0.00 0.04 0.00 0.00 0.00 0.00 0.19 0.00 0.00 0.00
2 0.38 0.00 0.00 0.00 0.45 0.28 0.16 0.00 0.03 0.29 0.43 0.00 0.00 0.25 0.26 0.00 0.21 0.00 0.12 0.43 0.02 0.00 0.00 0.00 0.00 0.00 0.13 0.00 0.00 0.00
3 0.26 0.00 0.00 0.19 0.08 0.07 0.31 0.00 0.07 0.51 0.14 0.01 0.00 0.01 0.00 0.00 0.14 0.00 0.00 0.00 0.00 0.09 0.00 0.00 0.00 0.25 0.23 0.01 0.00 0.02
4 0.08 0.00 0.19 0.00 0.09 0.29 0.08 0.00 0.46 0.00 0.23 0.02 0.00 0.00 0.00 0.13 0.00 0.00 0.08 0.18 0.09 0.13 0.00 0.00 0.11 0.06 0.06 0.01 0.00 0.04
5 0.31 0.45 0.08 0.09 0.00 0.42 0.00 0.01 0.00 0.20 0.31 0.00 0.00 0.25 0.14 0.00 0.00 0.00 0.00 0.26 0.00 0.05 0.00 0.00 0.00 0.00 0.20 0.00 0.00 0.00
6 0.40 0.28 0.07 0.29 0.42 0.00 0.00 0.00 0.22 0.21 0.11 0.00 0.00 0.00 0.02 0.07 0.00 0.00 0.10 0.53 0.10 0.05 0.00 0.00 0.07 0.00 0.23 0.00 0.00 0.00
7 0.51 0.16 0.31 0.08 0.00 0.00 0.00 0.00 0.10 0.18 0.29 0.00 0.00 0.00 0.12 0.00 0.35 0.00 0.00 0.00 0.00 0.04 0.00 0.00 0.00 0.04 0.00 0.00 0.00 0.00
8 0.00 0.00 0.00 0.00 0.01 0.00 0.00 0.00 0.00 0.00 0.00 0.00 0.00 0.01 0.01 0.00 0.00 0.67 0.00 0.00 0.00 0.00 0.00 0.06 0.01 0.00 0.00 0.58 0.00 0.03
9 0.00 0.03 0.07 0.46 0.00 0.22 0.10 0.00 0.00 0.00 0.13 0.00 0.00 0.00 0.02 0.10 0.00 0.00 0.13 0.14 0.13 0.05 0.00 0.00 0.08 0.05 0.00 0.00 0.00 0.00
10 0.38 0.29 0.51 0.00 0.20 0.21 0.18 0.00 0.00 0.00 0.00 0.00 0.00 0.10 0.00 0.00 0.22 0.00 0.09 0.36 0.00 0.00 0.00 0.00 0.00 0.18 0.31 0.00 0.00 0.00
11 0.24 0.43 0.14 0.23 0.31 0.11 0.29 0.00 0.13 0.00 0.00 0.00 0.00 0.16 0.31 0.00 0.12 0.00 0.02 0.00 0.02 0.10 0.00 0.00 0.00 0.04 0.04 0.01 0.00 0.03
12 0.00 0.00 0.01 0.02 0.00 0.00 0.00 0.00 0.00 0.00 0.00 0.00 0.84 0.00 0.02 0.55 0.00 0.00 0.00 0.01 0.60 0.00 0.00 0.00 0.04 0.00 0.00 0.00 0.00 0.00
13 0.00 0.00 0.00 0.00 0.00 0.00 0.00 0.00 0.00 0.00 0.00 0.84 0.00 0.00 0.00 0.56 0.00 0.00 0.00 0.00 0.51 0.00 0.01 0.00 0.00 0.00 0.00 0.00 0.00 0.01
14 0.00 0.25 0.01 0.00 0.25 0.00 0.00 0.01 0.00 0.10 0.16 0.00 0.00 0.00 0.50 0.00 0.48 0.00 0.60 0.11 0.00 0.00 0.00 0.00 0.37 0.00 0.00 0.00 0.00 0.00
15 0.10 0.26 0.00 0.00 0.14 0.02 0.12 0.01 0.02 0.00 0.31 0.02 0.00 0.50 0.00 0.00 0.44 0.00 0.38 0.00 0.04 0.01 0.01 0.01 0.59 0.00 0.00 0.00 0.00 0.00
16 0.00 0.00 0.00 0.13 0.00 0.07 0.00 0.00 0.10 0.00 0.00 0.55 0.56 0.00 0.00 0.00 0.00 0.00 0.06 0.10 0.81 0.00 0.00 0.00 0.08 0.01 0.01 0.00 0.00 0.00
17 0.28 0.21 0.14 0.00 0.00 0.00 0.35 0.00 0.00 0.22 0.12 0.00 0.00 0.48 0.44 0.00 0.00 0.00 0.57 0.10 0.00 0.00 0.00 0.00 0.36 0.00 0.00 0.00 0.00 0.00
18 0.00 0.00 0.00 0.00 0.00 0.00 0.00 0.67 0.00 0.00 0.00 0.00 0.00 0.00 0.00 0.00 0.00 0.00 0.00 0.00 0.00 0.19 0.19 0.25 0.00 0.00 0.00 0.85 0.00 0.00
19 0.00 0.12 0.00 0.08 0.00 0.10 0.00 0.00 0.13 0.09 0.02 0.00 0.00 0.60 0.38 0.06 0.57 0.00 0.00 0.22 0.07 0.00 0.00 0.00 0.56 0.00 0.00 0.00 0.00 0.00
20 0.25 0.43 0.00 0.18 0.26 0.53 0.00 0.00 0.14 0.36 0.00 0.01 0.00 0.11 0.00 0.10 0.10 0.00 0.22 0.00 0.07 0.00 0.00 0.00 0.09 0.01 0.16 0.00 0.00 0.00
21 0.00 0.02 0.00 0.09 0.00 0.10 0.00 0.00 0.13 0.00 0.02 0.60 0.51 0.00 0.04 0.81 0.00 0.00 0.07 0.07 0.00 0.00 0.00 0.00 0.08 0.00 0.00 0.00 0.00 0.00
22 0.04 0.00 0.09 0.13 0.05 0.05 0.04 0.00 0.05 0.00 0.10 0.00 0.00 0.00 0.01 0.00 0.00 0.19 0.00 0.00 0.00 0.00 0.75 0.77 0.01 0.31 0.28 0.20 0.04 0.02
23 0.00 0.00 0.00 0.00 0.00 0.00 0.00 0.00 0.00 0.00 0.00 0.00 0.01 0.00 0.01 0.00 0.00 0.19 0.00 0.00 0.00 0.75 0.00 0.77 0.01 0.26 0.23 0.20 0.10 0.13
24 0.00 0.00 0.00 0.00 0.00 0.00 0.00 0.06 0.00 0.00 0.00 0.00 0.00 0.00 0.01 0.00 0.00 0.25 0.00 0.00 0.00 0.77 0.77 0.00 0.01 0.27 0.23 0.21 0.04 0.02
25 0.00 0.00 0.00 0.11 0.00 0.07 0.00 0.01 0.08 0.00 0.00 0.04 0.00 0.37 0.59 0.08 0.36 0.00 0.56 0.09 0.08 0.01 0.01 0.01 0.00 0.01 0.01 0.00 0.00 0.00
26 0.00 0.00 0.25 0.06 0.00 0.00 0.04 0.00 0.05 0.18 0.04 0.00 0.00 0.00 0.00 0.01 0.00 0.00 0.00 0.01 0.00 0.31 0.26 0.27 0.01 0.00 0.73 0.00 0.00 0.00
27 0.19 0.13 0.23 0.06 0.20 0.23 0.00 0.00 0.00 0.31 0.04 0.00 0.00 0.00 0.00 0.01 0.00 0.00 0.00 0.16 0.00 0.28 0.23 0.23 0.01 0.73 0.00 0.00 0.00 0.00
28 0.00 0.00 0.01 0.01 0.00 0.00 0.00 0.58 0.00 0.00 0.01 0.00 0.00 0.00 0.00 0.00 0.00 0.85 0.00 0.00 0.00 0.20 0.20 0.21 0.00 0.00 0.00 0.00 0.01 0.08
29 0.00 0.00 0.00 0.00 0.00 0.00 0.00 0.00 0.00 0.00 0.00 0.00 0.00 0.00 0.00 0.00 0.00 0.00 0.00 0.00 0.00 0.04 0.10 0.04 0.00 0.00 0.00 0.01 0.00 0.10
30 0.00 0.00 0.02 0.04 0.00 0.00 0.00 0.03 0.00 0.00 0.03 0.00 0.01 0.00 0.00 0.00 0.00 0.00 0.00 0.00 0.00 0.02 0.13 0.02 0.00 0.00 0.00 0.08 0.10 0.00
1 2 3 4 5 6 7 8 9 10 11 12 13 14 15 16 17 18 19 20 21 22 23 24 25 26 27 28 29 30
0.80
0.70
0.60
0.50
0.40
0.30
0.20
0.10
