# Supplementary material for: Identifying differentially coexpressed module during HIV disease progression: A multiobjective approach
Source: Sci Rep. 2017 Mar 7;7:86. doi: 10.1038/s41598-017-00090-2 (PMC5428367; doi:10.1038/s41598-017-00090-2)
Supplement: Supplementary file 3 — Supplementary_table2 [file 41598_2017_90_MOESM3_ESM.pdf]

Identifying differentially coexpressed module during HIV disease progression: A multiobjective approach.

Sumanta Ray[1,*] **and Ujjwal Maulik**[2]
[1]Department of Computer Science and Engineering, Aliah University, Kolkata-700156, India
[2]Department of Computer Science and Engineering, Jadavpur University, Kolkata-700108, India
[*]sumantababai86@gmail.com

hsa-miR-221-3p	ABHD3
hsa-miR-3176	ACTB
hsa-miR-197-3p	ACTG1
hsa-miR-124-3p	ACTR3
hsa-miR-196a-5p	AHSA1
hsa-miR-3144-3p	ATM
hsa-miR-27a-3p	BMI1
hsa-miR-193b-3p	CCT2
hsa-miR-92a-3p	CDV3
hsa-miR-484	CMPK1
hsa-miR-361-5p	CYCS
hsa-miR-10a-5p	DNAJB1
hsa-miR-7-5p	EHD1
hsa-miR-92a-3p	ERAP1
hsa-let-7c-5p	EWSR1
hsa-miR-222-3p	FAM53C
hsa-miR-30a-5p	GLIPR1
hsa-miR-335-5p	GPR34
hsa-miR-197-3p	H2AFZ
hsa-miR-92a-3p	HEATR1
hsa-miR-34a-5p	HIST2H2AA4
hsa-miR-615-3p	HLA-A
hsa-miR-615-3p	HLA-B
hsa-miR-3187-3p	HN1
hsa-let-7a-5p	HUWE1
hsa-miR-17-3p	KPNA2
hsa-miR-15a-5p	MAPK6
hsa-miR-133a-3p	NR4A2
hsa-let-7c-5p	NUDT21
hsa-miR-30c-2-3p	PITPNB
hsa-miR-1249	RBMS1
hsa-miR-196a-5p	RBMX
hsa-miR-31-5p	RPL37A
hsa-let-7a-5p	RPS15A
hsa-miR-1260b	RPS6
hsa-miR-17-5p	42249
hsa-miR-148a-3p	SLC2A3
hsa-let-7b-5p	SNRPE
hsa-miR-197-3p	SOD1
hsa-miR-1229-3p	SRP72
hsa-miR-615-3p	STAT1
hsa-miR-1180-3p	SYNRG
hsa-miR-3176	TAGLN2
hsa-miR-16-5p	TGOLN2
hsa-miR-17-5p	TMSB10
hsa-miR-30c-5p	TRA2A
hsa-miR-17-5p	TRA2B

hsa-miR-93-5p	TRAM1
hsa-miR-186-5p	TSPYL4
hsa-miR-106b-5p	UGP2
hsa-miR-98-5p	AASDHPPT
hsa-miR-615-3p	ACADVL
hsa-miR-484	ADAM10
hsa-miR-20a-5p	ADSS
hsa-miR-324-5p	ANAPC13
hsa-miR-320a	ANP32A
hsa-miR-26b-5p	ANXA1
hsa-miR-16-5p	ATP6V0E1
hsa-miR-221-3p	ATP6V1E1
hsa-miR-93-5p	B2M
hsa-let-7b-5p	BAZ1A
hsa-let-7b-5p	BRD2
hsa-let-7b-5p	C6orf62
hsa-miR-1	CALM1
hsa-miR-149-5p	CALR
hsa-miR-3934-5p	CAPZB
hsa-miR-93-5p	CBX3
hsa-miR-92a-3p	CCNI
hsa-miR-16-5p	CCT8
hsa-miR-505-3p	CD47
hsa-miR-484	CD97
hsa-miR-16-5p	CIB1
hsa-miR-23b-3p	CLTA
hsa-miR-98-5p	COQ10B
hsa-miR-92a-3p	COX4I1
hsa-miR-148b-3p	COX7A2
hsa-miR-18a-5p	CREBL2
hsa-miR-671-5p	CRLF3
hsa-miR-128-3p	DCK
hsa-miR-30c-5p	DDOST
hsa-miR-378c	DDX17
hsa-miR-99a-5p	DDX3X
hsa-miR-1260b	DDX3Y
hsa-let-7e-5p	DHX15
hsa-miR-18b-5p	DPM1
hsa-miR-455-5p	DRG1
hsa-miR-92a-3p	DYRK1A
hsa-miR-149-5p	EDF1
hsa-miR-149-5p	EEF1D
hsa-miR-92a-3p	EIF2B2
hsa-miR-17-5p	EIF4G2
hsa-miR-100-5p	EIF5AL1
hsa-miR-124-3p	FAM65B
hsa-miR-16-5p	FAM96B

hsa-miR-505-3p	FIS1
hsa-miR-1	FOLR1
hsa-miR-16-5p	GABARAPL1
hsa-miR-92a-3p	GAK
hsa-miR-18a-5p	GCH1
hsa-miR-222-3p	GDI1
hsa-miR-15a-5p	GDI2
hsa-miR-16-5p	GOLGA7
hsa-miR-17-3p	H2AFV
hsa-let-7b-5p	HADHA
hsa-miR-92a-3p	HLA-E
hsa-miR-148a-3p	HLA-G
hsa-miR-16-5p	HMGN1
hsa-miR-92a-3p	HSD17B10
hsa-miR-3943	HSP90AA1
hsa-miR-92a-3p	HSP90AB1
hsa-miR-26b-5p	ITGB2
hsa-miR-1260b	JMJD6
hsa-miR-186-5p	JOSD1
hsa-miR-186-5p	JUND
hsa-miR-484	KARS
hsa-miR-92a-3p	KPNB1
hsa-miR-335-5p	LAPTM5
hsa-miR-320a	LMNB1
hsa-miR-615-3p	LRRC8D
hsa-miR-26b-5p	LSM1
hsa-miR-125b-5p	LUC7L3
hsa-miR-92a-3p	MAPK1IP1L
hsa-miR-1260b	MTCH1
hsa-miR-30c-5p	MTDH
hsa-miR-93-5p	N4BP1
hsa-miR-18a-3p	NAA16
hsa-miR-328-3p	NDUFA1
hsa-miR-18a-3p	NDUFB3
hsa-miR-100-5p	NDUFC2
hsa-let-7a-5p	NFATC2IP
hsa-miR-92a-3p	NHP2L1
hsa-miR-26b-5p	NSL1
hsa-miR-17-5p	OPTN
hsa-miR-92a-3p	OSBPL8
hsa-let-7b-5p	OTUB1
hsa-let-7b-5p	PCBP2
hsa-let-7b-5p	PDS5A
hsa-miR-20a-5p	PGK1
hsa-miR-185-5p	PHACTR2
hsa-miR-25-3p	PHB2
hsa-miR-125b-5p	PMAIP1

hsa-miR-320a	PNN
hsa-miR-124-3p	POLR2J
hsa-miR-92a-3p	POLR2L
hsa-miR-17-5p	PPP1R15A
hsa-miR-31-3p	PPP2R5C
hsa-miR-10b-5p	PPP3CB
hsa-miR-378a-5p	PRMT1
hsa-miR-10a-5p	PSMD13
hsa-miR-196a-5p	PSMD8
hsa-miR-378a-3p	PTMA
hsa-let-7a-5p	RAD21
hsa-miR-125b-5p	RASGRP1
hsa-miR-20a-5p	RBM10
hsa-miR-22-3p	RGS2
hsa-miR-124-3p	RHOG
hsa-miR-186-5p	RNF113A
hsa-miR-7-5p	RNF114
hsa-miR-423-5p	RNF139
hsa-miR-93-5p	RNF44
hsa-miR-100-5p	S100A10
hsa-miR-196a-5p	SAP18
hsa-miR-1260b	SCP2
hsa-miR-30c-5p	SDF2L1
hsa-miR-34a-5p	42254
hsa-miR-335-5p	SERPINB9
hsa-miR-222-3p	SLC25A36
hsa-miR-18a-5p	SMCHD1
hsa-miR-100-5p	SNRNP27
hsa-miR-375	SP110
hsa-miR-221-3p	SRP68
hsa-miR-27a-3p	SUN2
hsa-miR-29a-3p	SYNCRIP
hsa-miR-92a-3p	TFG
hsa-miR-16-5p	TFRC
hsa-miR-98-5p	TNFSF10
hsa-miR-101-3p	TOR1AIP1
hsa-let-7b-5p	TPM4
hsa-miR-192-5p	TRAF3IP3
hsa-miR-26b-5p	TRIM22
hsa-miR-92a-3p	U2AF2
hsa-miR-26a-5p	UBA2
hsa-miR-26b-5p	UBE2E1
hsa-miR-149-5p	UBE2N
hsa-miR-30a-5p	UFM1
hsa-let-7b-5p	VPS28
hsa-let-7b-5p	WBP11
hsa-miR-335-5p	XAF1

First column indicates miRNA names and second colum
by those miRNAs. The red highlighted rows are TFs regu
miRNA.

hsa-miR-100-5p	XRCC6
hsa-let-7b-5p	YTHDC1
hsa-miR-221-3p	YWHAB
hsa-miR-221-3p	YY1
hsa-miR-96-5p	ZC3H15
hsa-miR-346	ZFP36
hsa-miR-24-3p	ADD3
hsa-let-7b-5p	CCND3
hsa-miR-23a-3p	CD302
hsa-let-7e-5p	CELF2
hsa-miR-346	CNN2
hsa-miR-320a	COX6B1
hsa-miR-106b-5p	CTDSP2
hsa-miR-769-5p	DDX24
hsa-miR-138-5p	EID1
hsa-miR-16-5p	EIF3CL
hsa-let-7b-5p	HIF1A
hsa-miR-92b-3p	KDM2A
hsa-let-7a-5p	KDM6B
hsa-miR-505-3p	LIMS1
hsa-miR-1260b	MCM7
hsa-miR-744-5p	MEX3C
hsa-miR-146a-5p	MX2
hsa-miR-25-3p	PARL
hsa-miR-16-5p	PDE3B
hsa-miR-124-3p	PNPLA2
hsa-miR-26b-5p	POLR2G
hsa-miR-25-3p	PRKAR1A
hsa-miR-25-3p	PSMB4
hsa-miR-93-5p	PSMD4
hsa-miR-10a-5p	PWP1
hsa-miR-17-5p	QARS
hsa-let-7e-5p	RAP1A
hsa-miR-26a-5p	SETD2
hsa-miR-1307-3p	SF3B5
hsa-miR-423-3p	SLC25A6
hsa-miR-1260b	SP3
hsa-miR-378a-3p	STXBP3
hsa-let-7e-5p	SUZ12
hsa-miR-324-5p	TBCB
hsa-miR-590-3p	TMEM123
hsa-miR-324-5p	UBE2I
hsa-miR-196a-5p	VDAC3
hsa-miR-18a-5p	ZFP36L2
hsa-let-7b-5p	ZNF207
hsa-miR-26a-5p	AURKAIP1
hsa-miR-186-5p	CUEDC2

hsa-miR-18a-5p	DDX5
hsa-let-7b-5p	HSPA8
hsa-miR-18a-3p	ST6GAL1
hsa-miR-106b-5p	YTHDF2
hsa-miR-365a-3p	ANKRD12
hsa-miR-1	ISG20
hsa-miR-26b-5p	KAT2B
hsa-miR-92a-3p	PDAP1
hsa-miR-23a-3p	CSDE1
hsa-miR-484	N4BP2L2
hsa-miR-17-5p	RPL37
hsa-let-7e-5p	MATR3
hsa-miR-98-5p	C19orf53
hsa-let-7b-5p	MAT2B
hsa-miR-26b-5p	CXCR4
hsa-miR-149-5p	FDFT1
hsa-miR-197-3p	GSTO1
hsa-miR-26b-5p	IFI16
hsa-miR-17-5p	LAPTM4A
hsa-miR-423-3p	LSM4
hsa-miR-16-5p	NUDT3
hsa-miR-19b-3p	PRKACB
hsa-miR-484	RING1
hsa-miR-615-3p	SERINC3
hsa-miR-331-3p	YME1L1
hsa-miR-99a-5p	PUM1
hsa-miR-335-5p	CD37
hsa-miR-92a-3p	SNRNP70
hsa-miR-30a-5p	ERP44
hsa-miR-423-3p	LYPLA2
hsa-miR-769-3p	TSPAN14
hsa-miR-98-5p	STK17B

ın reoresents genes regulated
ulated by the corresponding
