# Supplementary material for: Identifying differentially coexpressed module during HIV disease progression: A multiobjective approach
Source: Sci Rep. 2017 Mar 7;7:86. doi: 10.1038/s41598-017-00090-2 (PMC5428367; doi:10.1038/s41598-017-00090-2)
Supplement: Supplementary file 4 — supplementary_table3 [file 41598_2017_90_MOESM4_ESM.pdf]

Identifying differentially coexpressed module during HIV disease progression: A multiobjective approach.

Sumanta Ray[1,*] **and Ujjwal Maulik**[2]
[1]Department of Computer Science and Engineering, Aliah University, Kolkata-700156, India
[2]Department of Computer Science and Engineering, Jadavpur University, Kolkata-700108, India
[*]sumantababai86@gmail.com

hsa-miR-221-3p'	'ABHD3'
'hsa-miR-92a-3p'	'CDV3'
'hsa-miR-92a-3p'	'HEATR1'
'hsa-miR-615-3p'	'HLA-A'
'hsa-miR-615-3p'	'HLA-B'
'hsa-let-7c-5p'	'NUDT21'
'hsa-miR-1260b'	'RPS6'
'hsa-let-7b-5p'	'SNRPE'
'hsa-miR-186-5p'	'TSPYL4'
'hsa-miR-615-3p'	'ACADVL'
'hsa-miR-20a-5p'	'ADSS'
'hsa-miR-221-3p'	'ATP6V1E1'
'hsa-let-7b-5p'	'BAZ1A'
'hsa-let-7b-5p'	'BRD2'
'hsa-let-7b-5p'	'C6orf62'
'hsa-miR-149-5p'	'CALR'
'hsa-miR-92a-3p'	'CCNI'
'hsa-miR-92a-3p'	'COX4I1'
'hsa-miR-1260b'	'DDX3Y'
'hsa-let-7e-5p'	'DHX15'
'hsa-miR-92a-3p'	'DYRK1A'
'hsa-miR-149-5p'	'EEF1D'
'hsa-miR-92a-3p'	'EIF2B2'
'hsa-miR-100-5p'	'EIF5AL1'
'hsa-miR-92a-3p'	'GAK'
'hsa-miR-18a-5p'	'GCH1'
'hsa-miR-92a-3p'	'HLA-E'
'hsa-miR-92a-3p'	'HSD17B10'
'hsa-miR-92a-3p'	'HSP90AB1'
'hsa-miR-1260b'	'JMJD6'
'hsa-miR-186-5p'	'JOSD1'
'hsa-miR-92a-3p'	'KPNB1'
'hsa-miR-615-3p'	'LRRC8D'
'hsa-miR-92a-3p'	'MAPK1IP1L'
'hsa-miR-1260b'	'MTCH1'
'hsa-miR-100-5p'	'NDUFC2'
'hsa-miR-92a-3p'	'NHP2L1'
'hsa-miR-92a-3p'	'OSBPL8'
'hsa-let-7b-5p'	'OTUB1'
'hsa-let-7b-5p'	'PCBP2'
'hsa-let-7b-5p'	'PDS5A'
'hsa-miR-20a-5p'	'PGK1'
'hsa-miR-100-5p'	'S100A10'
'hsa-miR-1260b'	'SCP2'
'hsa-miR-18a-5p'	'SMCHD1'
'hsa-miR-100-5p'	'SNRNP27'
'hsa-miR-221-3p'	'SRP68'

'hsa-miR-92a-3p'	'TFG'
'hsa-let-7b-5p'	'TPM4'
'hsa-miR-92a-3p'	'U2AF2'
'hsa-miR-149-5p'	'UBE2N'
'hsa-let-7b-5p'	'WBP11'
'hsa-let-7b-5p'	'YTHDC1'
'hsa-miR-221-3p'	'YWHAB'
'hsa-let-7b-5p'	'CCND3'
'hsa-let-7e-5p'	'CELF2'
'hsa-miR-346'	'CNN2'
'hsa-miR-1260b'	'MCM7'
'hsa-let-7e-5p'	'RAP1A'
'hsa-miR-186-5p'	'CUEDC2'
'hsa-miR-18a-5p'	'DDX5'
'hsa-miR-92a-3p'	'PDAP1'
'hsa-miR-615-3p'	'SERINC3'
